# Experimental and computational data on two-photon absorption and spectral deconvolution of the upper excited states of dye IR780

**DOI:** 10.1016/j.dib.2021.106752

**Published:** 2021-01-15

**Authors:** Luis Guillermo Mendoza-Luna, Cesar A. Guarin, Emmanuel Haro-Poniatowski, José Luis Hernández-Pozos

**Affiliations:** a Departamento de Física, Universidad Autónoma Metropolitana Iztapalapa, Av. San Rafael Atlixco No. 186 Col. Vicentina, C.P. 09340 México D.F., México; b Cátedras CONACYT - Departamento de Física, Universidad Autónoma Metropolitana Iztapalapa, Av. San Rafael Atlixco No. 186 Col. Vicentina, C.P. 09340 México D.F., México

**Keywords:** Two-photon absorption, Cyanine-type dyes, Upper excited states, Oscillator Strength, Non-linear properties of organic molecules, Optical properties calculated via DFT methods, Biomarkers

## Abstract

Evaluating candidates for novel materials with high nonlinear absorption properties for applications as biomarkers is a very important field of research. In this context, experimental and computational information on the two-photon absorption (TPA) properties of the dye IR780 is shown. The two-photon absorption data from 850 to 1000 nm for IR780 and other two well-known dyes, taken as reference, are presented. The experimental data were collected via an implementation of the two-photon induced fluorescence technique, while the quantum chemical data were produced by implementing DFT (Density-functional theory) methods. The data presented here supplement the paper “Two-photon absorption spectrum and characterization of the upper electronic states of dye IR780” by Guarin et al. (2021).

## Specifications Table

**Table:** 

Subject	Physical Sciences.
Specific subject area	Two-photon absorption, ultrafast spectroscopy, dyes as biomarkers.
Type of data	Tables and figures.
How data were acquired	A two-photon induced fluorescence (TPIF) scheme was implemented: an excitation beam was produced by an optical parametric amplifier (model TOPAS by Light Conversion) pumped by a regenerative amplifier (Legend Elite-DUO, Coherent Inc.) operating at 1 kHz. The amplifier produced 80 fs pulses, centered at 800 nm (spectral width of 20 nm) with an energy of 4.5 mJ per pulse. The TOPAS was tuned from 850 to 1000 nm and the power delivered to the sample was controlled with a λ/2 plate. The fluorescence of the samples was detected with an *f* = 30 cm Czerny-Turner monochromator fitted with a PMT (R12896, Hamamatsu Photonics) coupled to an oscilloscope (Tektronix TBS 1102B-EDU), where the signal was averaged over 64 samples.
Data format	Raw. Analyzed.
Parameters for data collection	The dyes used in this experiment were IR780, Rhodamines B and 6G in a methanol (HPLC-grade) solution with concentrations of 2.25 × 10^−5^ M, 7.65 × 10^−6^ M and 8.06 × 10^−6^ M, respectively. The excitation beam was focused with a microscope objective (NA=0.1, 4x). The energy of the excitation beam was varied between 0.002 and 0.35 μJ. UV–vis and luminescence spectra for IR780 in methanol were recorded in Perkin-Elmer spectrometers.
Description of data collection	The logarithm of the intensity of the fluorescence was plotted vs. the logarithm of the peak intensity of the excitation light. The deconvolutions and the fit of the Gaussian peaks were made with the Levenberg-Marquardt method implemented in Origin 2020b. The raw data and the fits have been supplied as Excel files (xlsx). Computational data were obtained from optimization and vertical transitions routines implemented in quantum-chemical programs Gaussian 09 and GAMESS-US, respectively.
Data source location	Femtosecond spectroscopy laboratory at the Physics Department of Universidad Autónoma Metropolitana-Iztapalapa, Mexico City, Mexico.
Data accessibility	Relevant data reported in this article and available at [1]. Repository name: Mendeley Data. Data identification number: https://doi.org/10.17632/2b3x7pgmfx.3 (https://data.mendeley.com/datasets/2b3x7pgmfx/3)
Related research article	Authors: Cesar A. Guarin, Luis Guillermo Mendoza-Luna, Emmanuel Haro-Poniatowski, José Luis Hernández-Pozos. Title: “Two photon absorption spectrum and characterization of the upper electronic states of the dye IR780”. Journal: Spectrochimica Acta Part A: Molecular and Biomolecular Spectroscopy, 249 (2021) 119291. https://doi.org/10.1016/j.saa.2020.119291

## Value of the Data

• These data show a measurement of the two-photon absorption band of the dye IR780.
• The data and associated calculations are useful for researchers interested in the nonlinear properties of cyanines as well as users looking into the potential of these substances as biomarkers excitable via IR radiation.
• The fits of the UV bands in the 1PA (one-photon absorption) spectrum can serve as reference for the fitting of the absorption spectra of other cyanines.

## 1 Data Description

The data presented here are experimental results to better understand the two-photon absorption processes involved in cyanine IR780 (2-[2-[2-Chloro-3-[(1,3-dihydro-3,3-dimethyl-1-propyl-2H-indol-2-ylidene)ethylidene]-1-cyclohexen-1-yl]ethenyl]-3,3-dimethyl-1-propylindolium iodide).

**Fig. 1:**
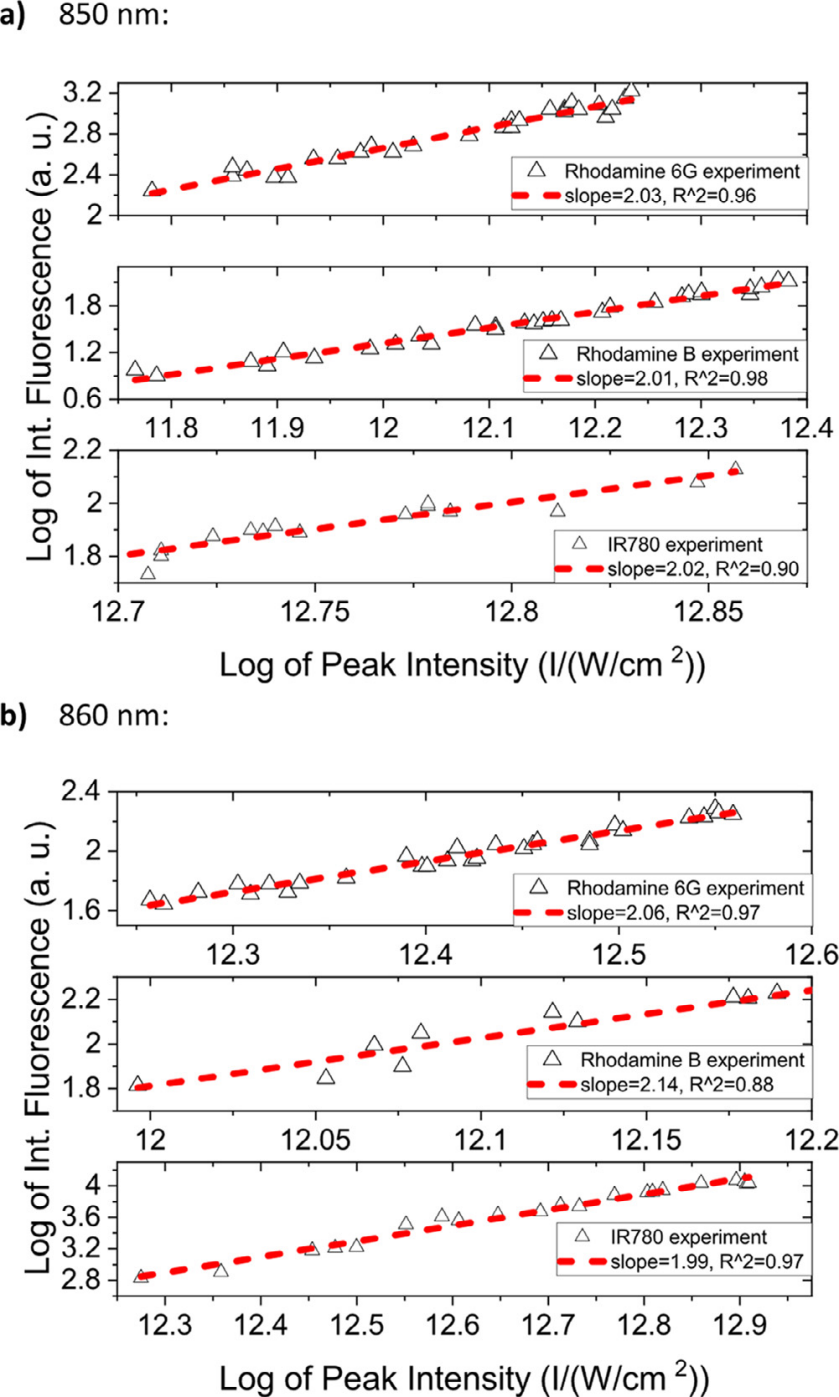

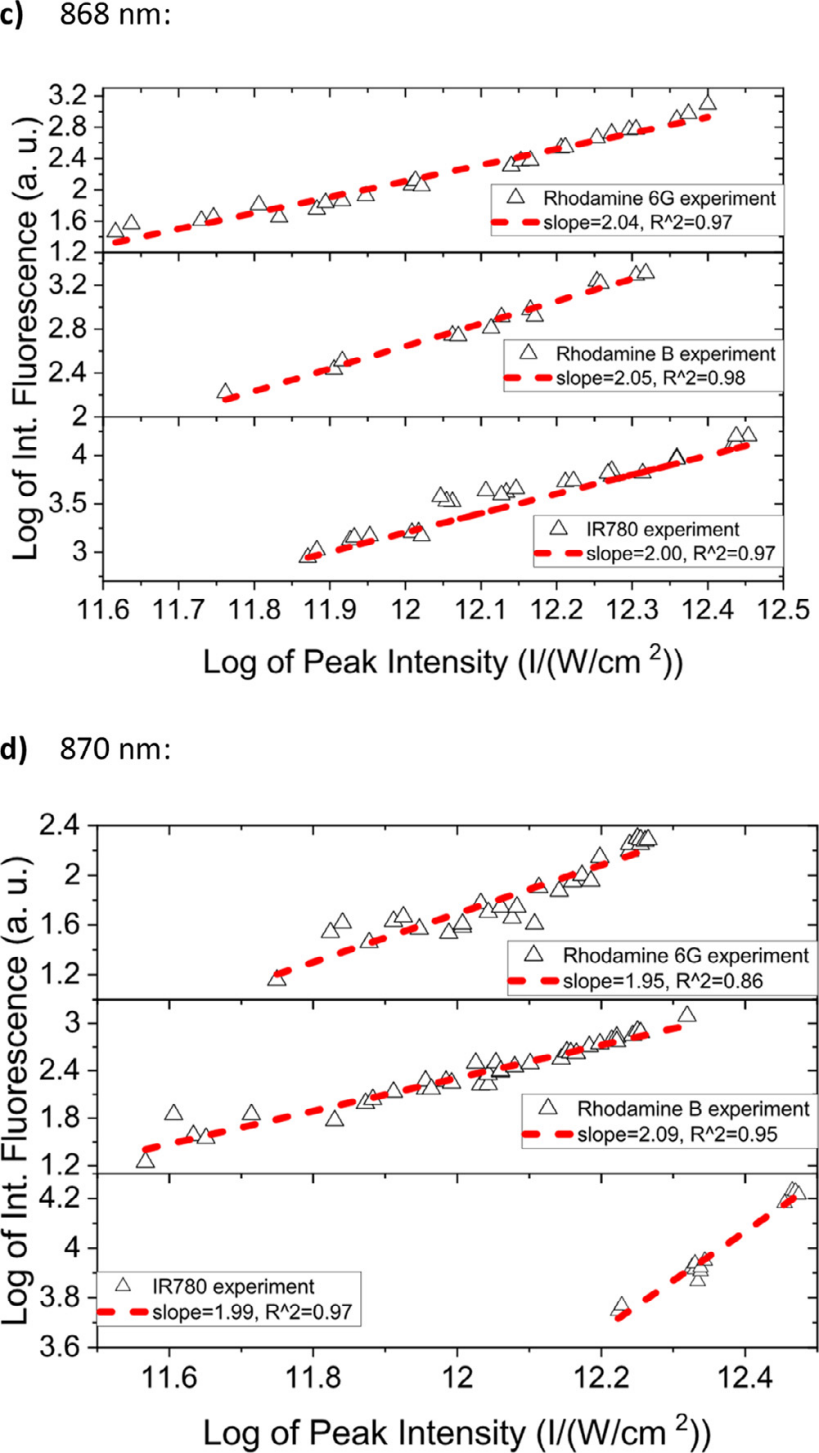

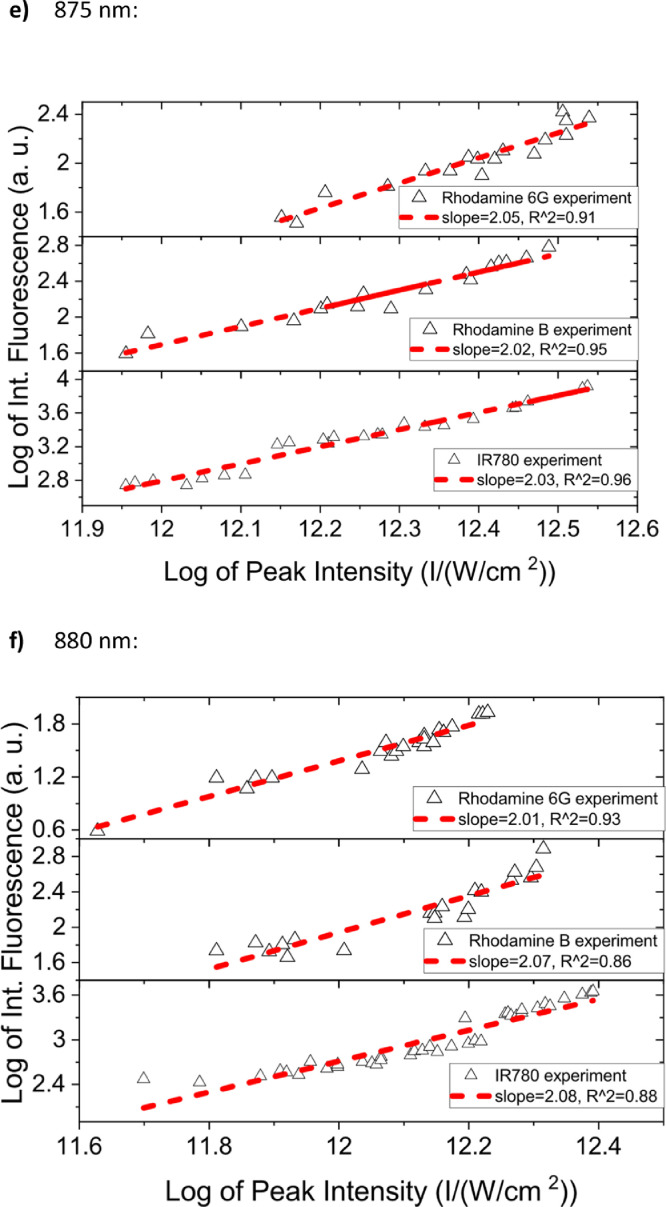

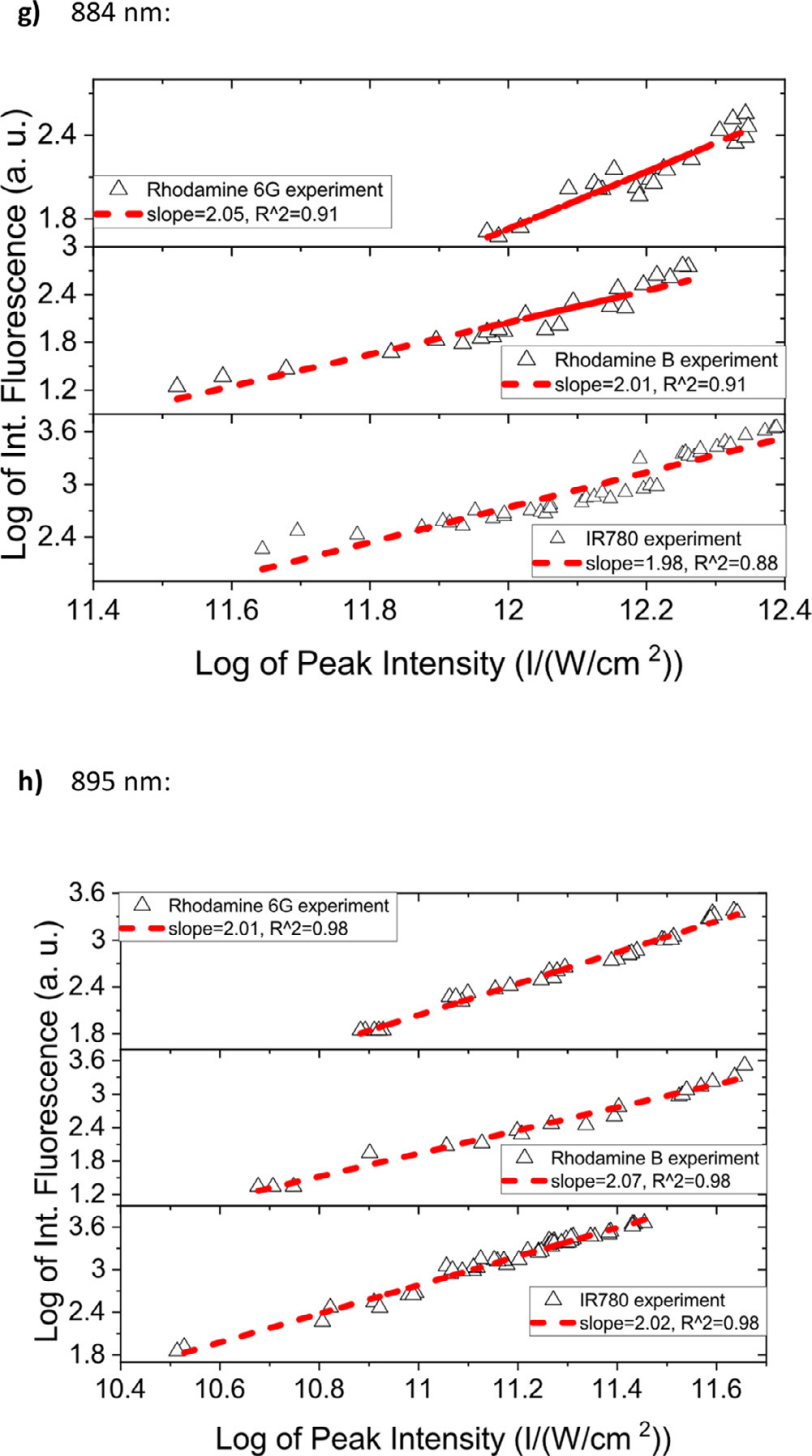

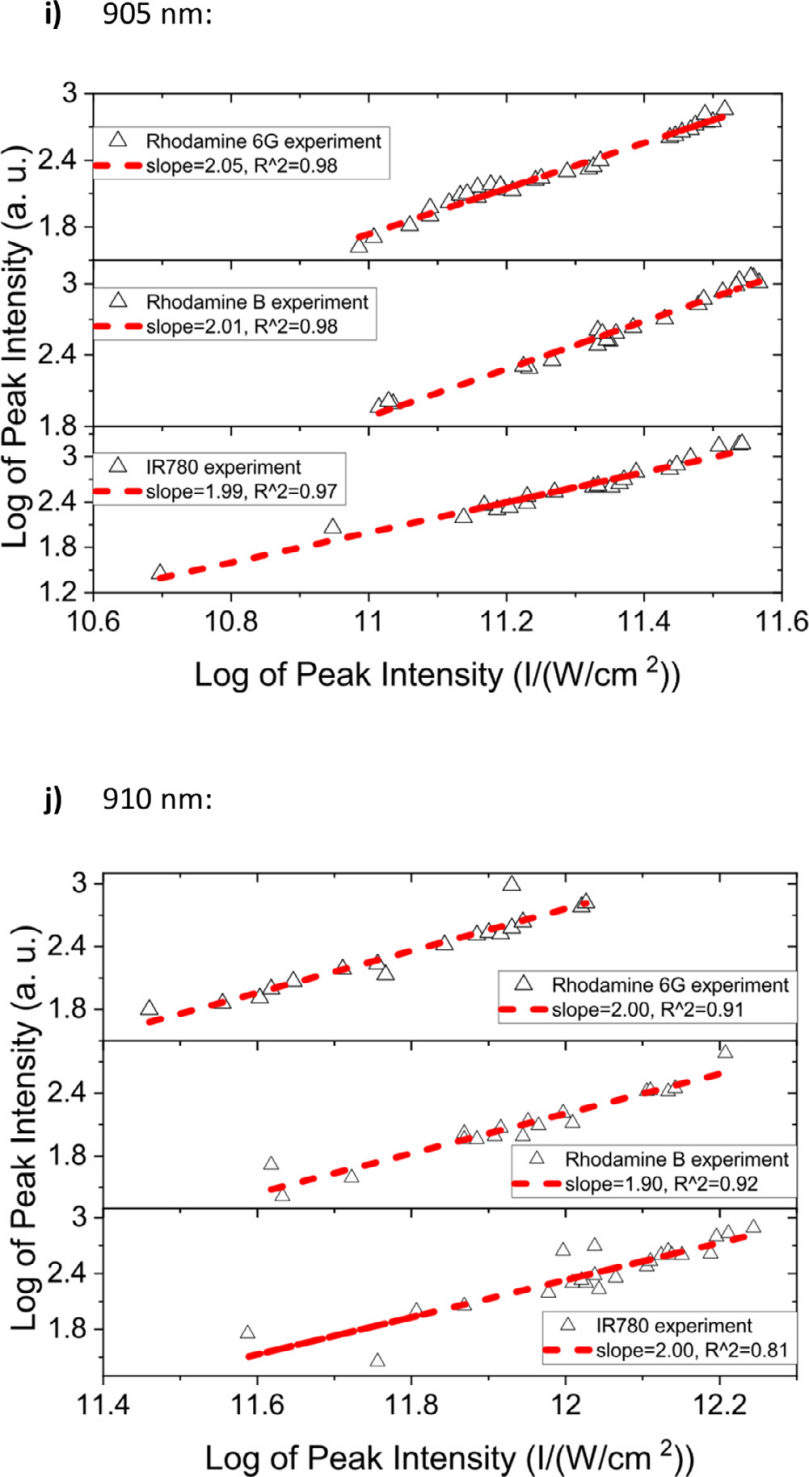

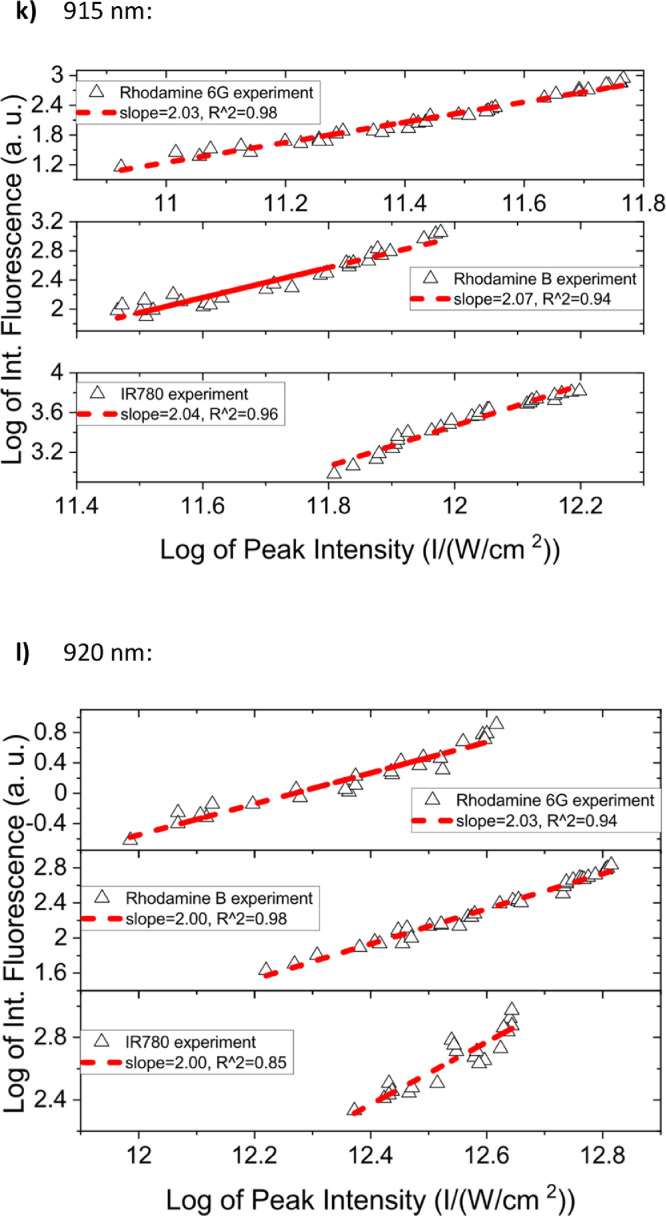

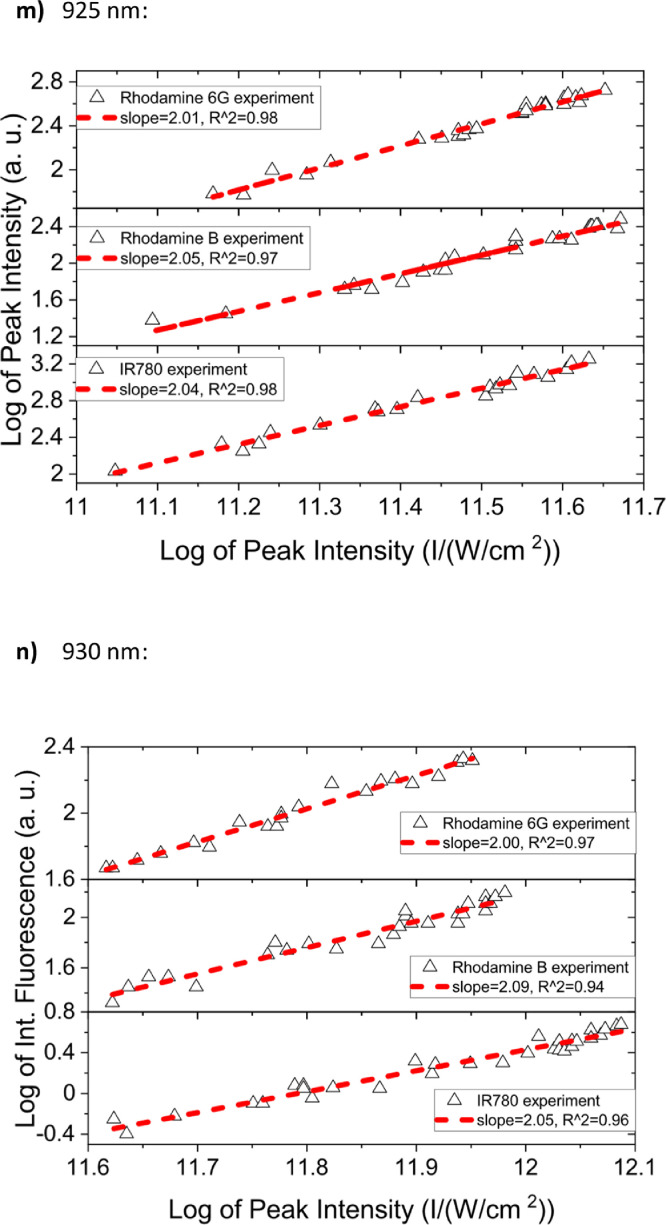

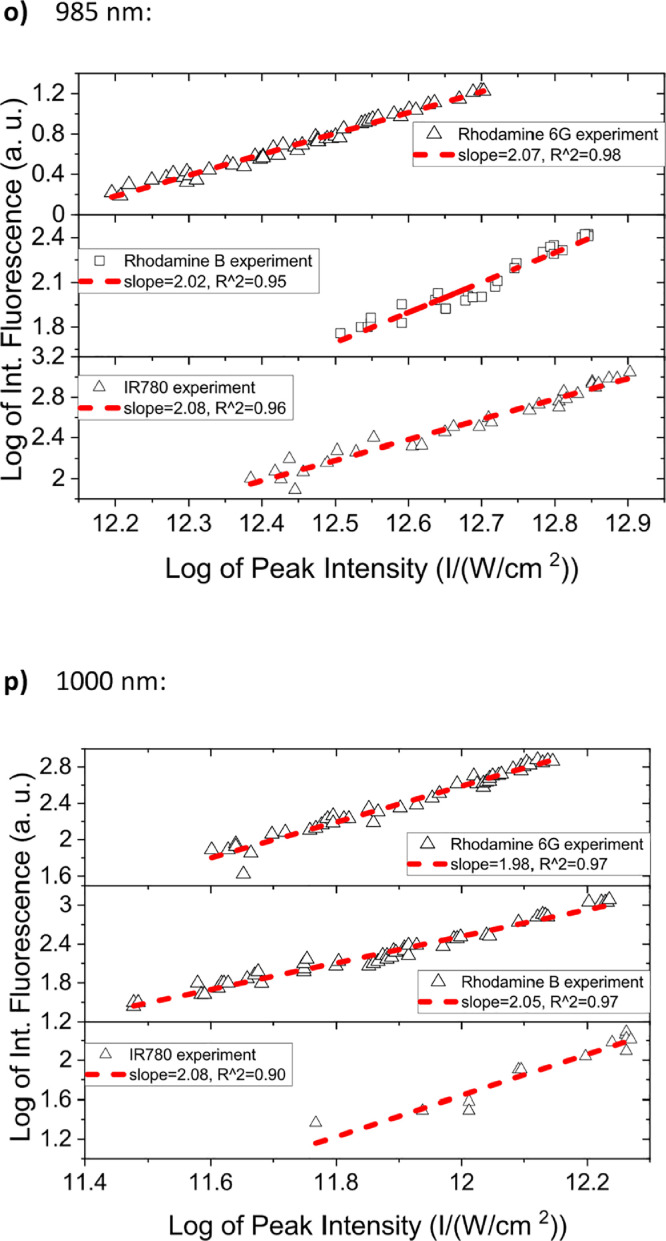
Set of graphs of the Log of the integrated fluorescence in methanol vs. the Log of the irradiation peak power for Rhodamine 6G, Rhodamine B and IR780 for several excitation wavelengths.

**Fig. 2:**
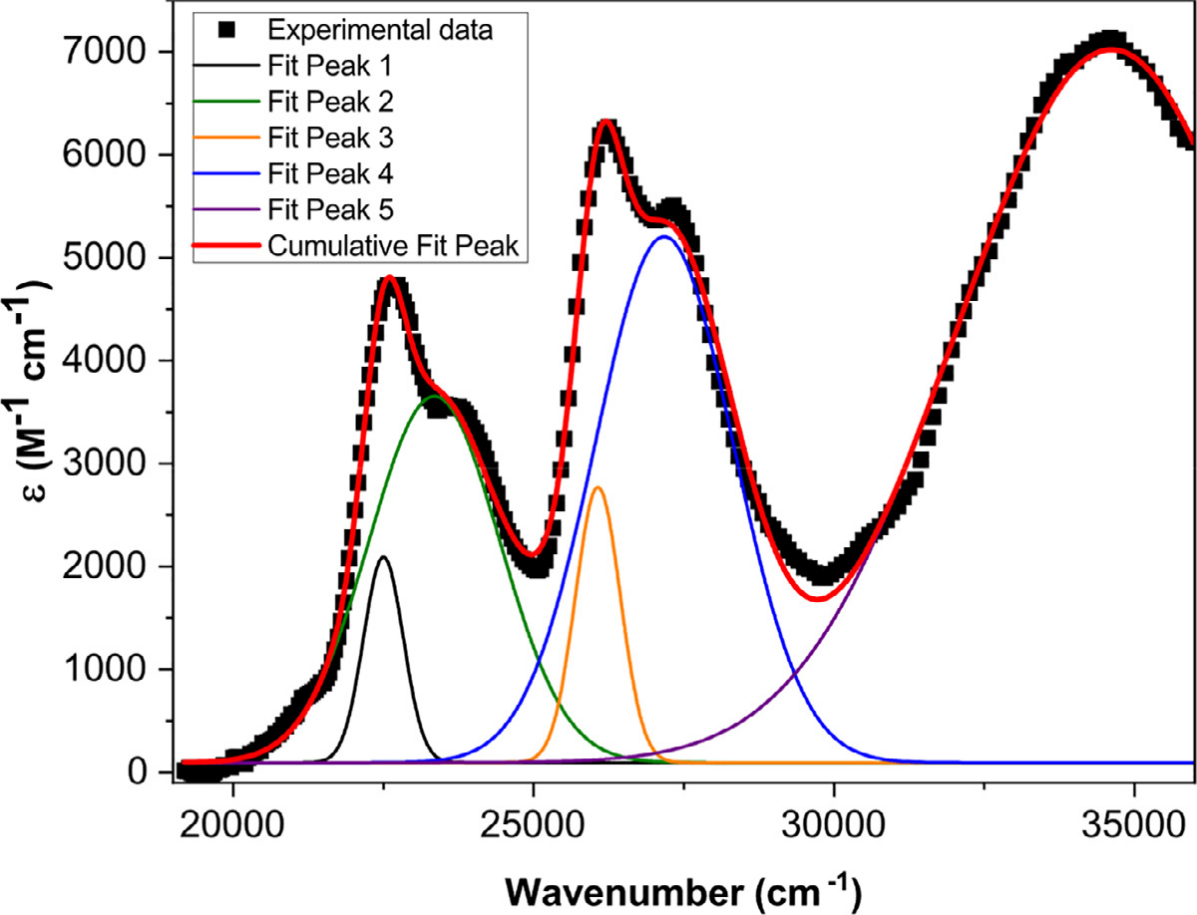
Curve fitting (5 Gaussian peaks) of the spectral bands of the upper states S_n_ of IR780.

**Fig. 3:**
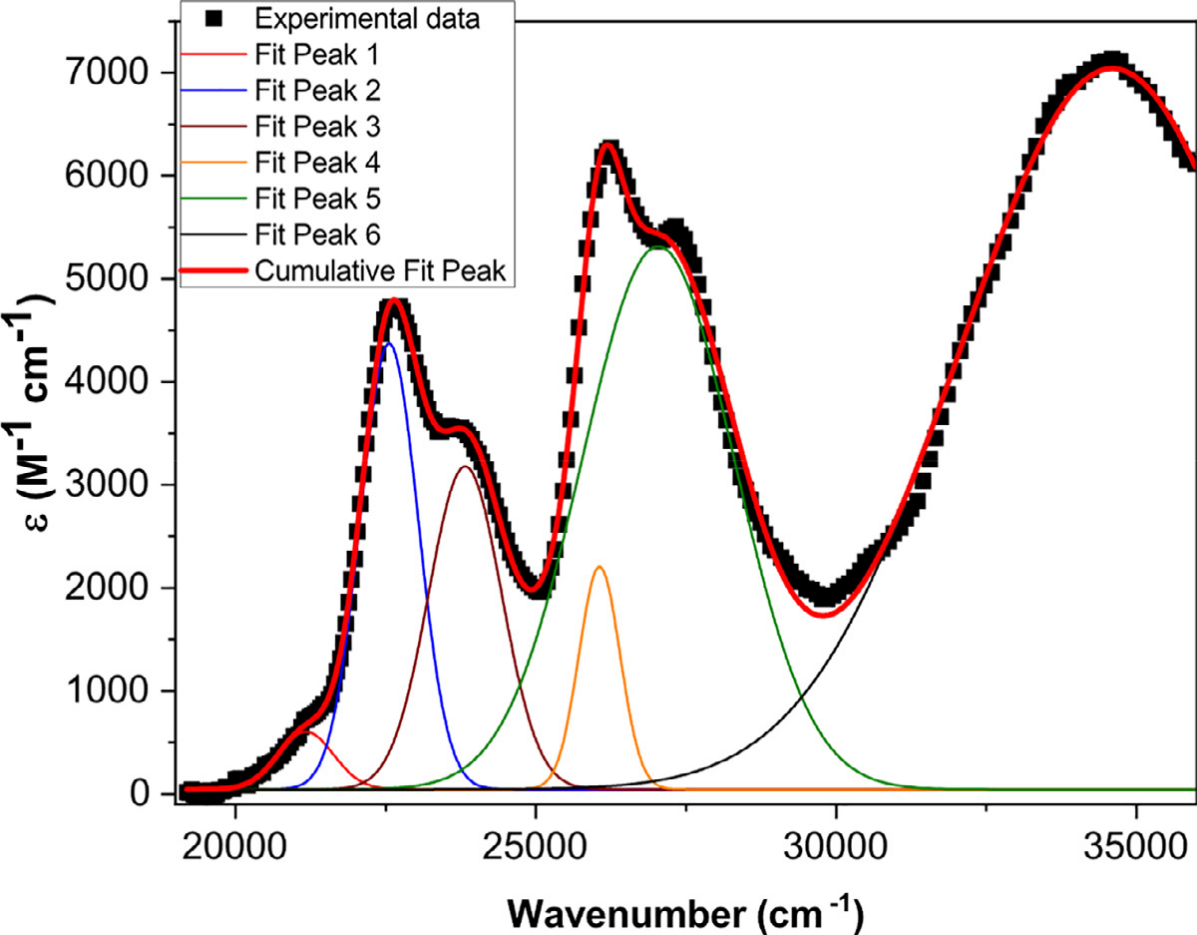
Curve fitting (6 Gaussian peaks) of the spectral bands of the upper states S_n_ of IR780.

**Fig. 4:**
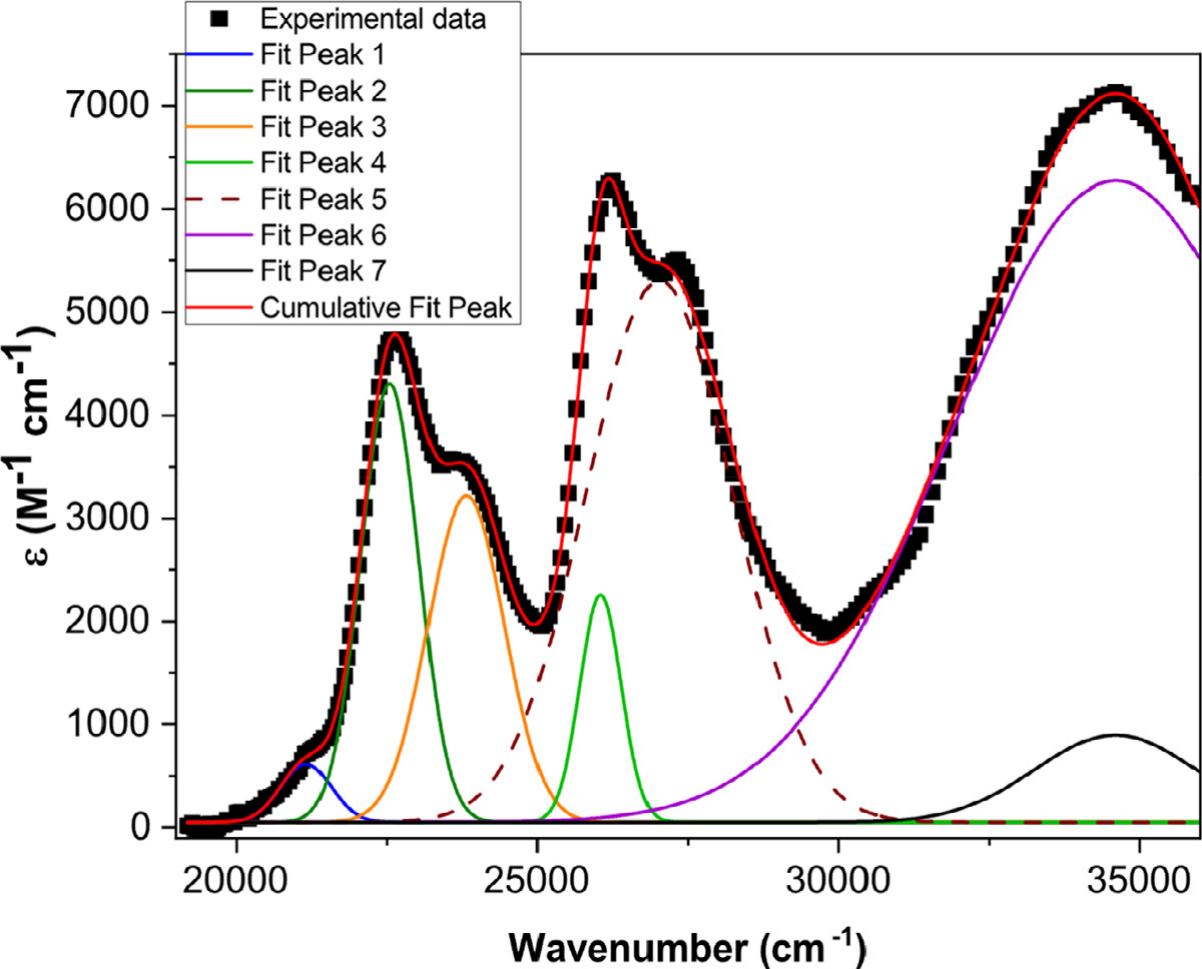
Curve fitting (7 Gaussian peaks) of the spectral bands of the upper states S_n_ of IR780.

**Fig. 5:**
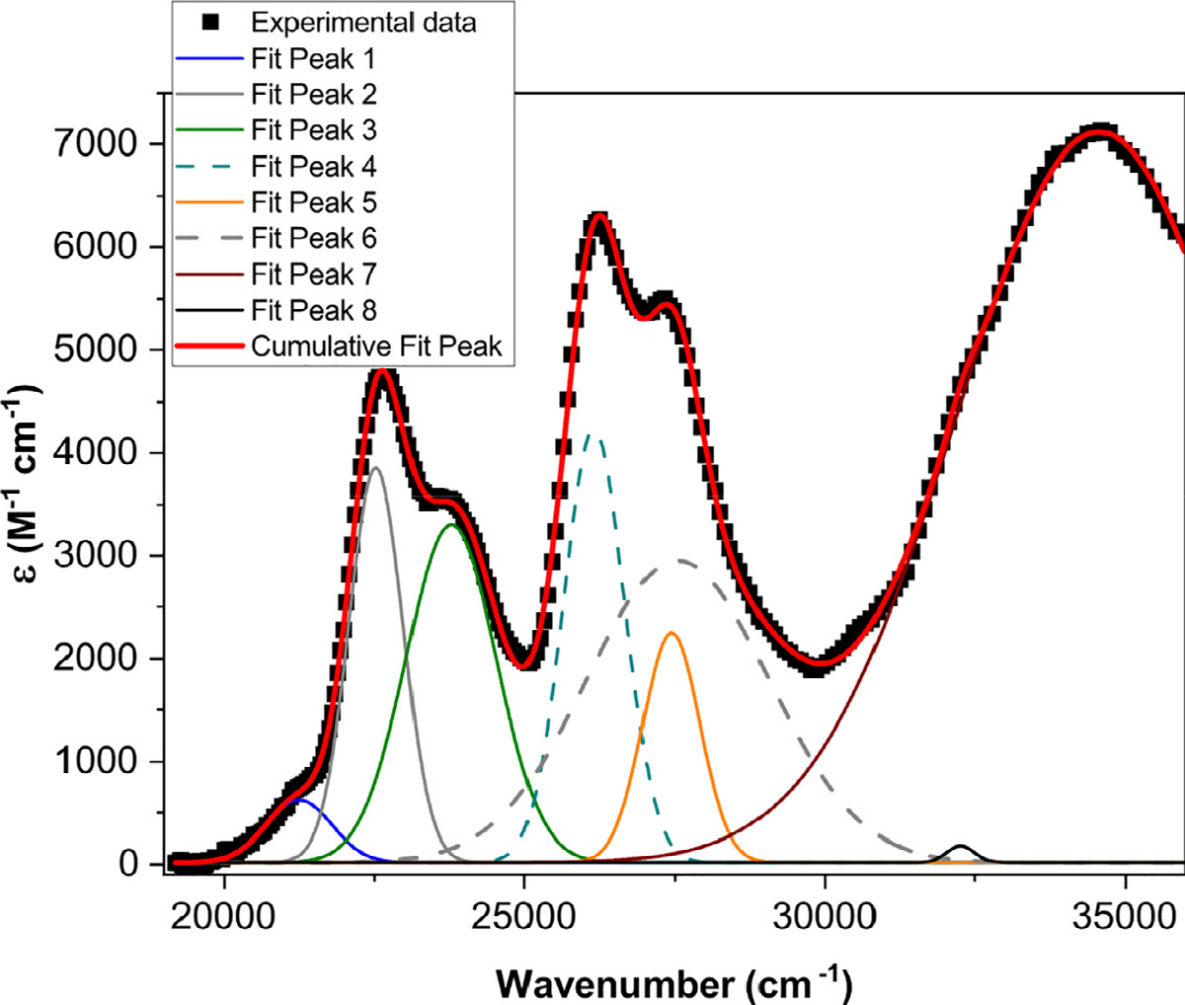
Curve fitting (8 Gaussian peaks) of the spectral bands of the upper states S_n_ of IR780.

**Fig. 6:**
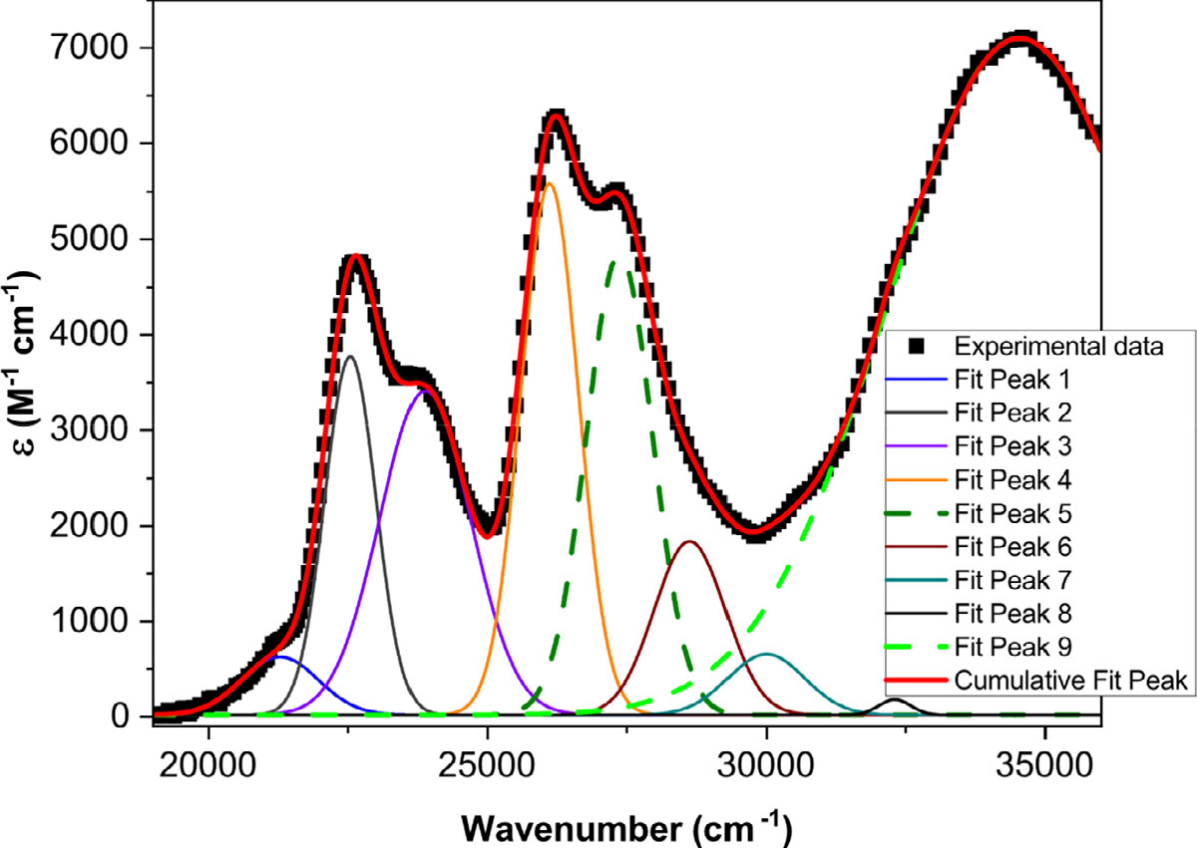
Curve fitting (9 Gaussian peaks) of the spectral bands of the upper states S_n_ of IR780.

**Fig. 7:**
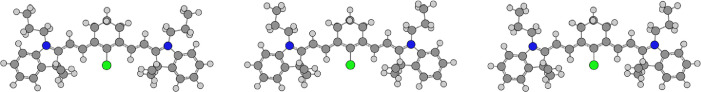
Optimized structures of molecule IR780 as per functionals PBE (left), PBE0 (center) and CAM-B3LYP (right).

Fig. 1 shows the quadratic dependence of the logarithm of the integrated fluorescence vs. the logarithm of the peak intensity of the pump beam for IR780, rhodamine B and rhodamine 6G; this is done for several wavelengths ranging from 850 to 1000 nm. Fig. 2, Fig. 3, Fig. 4, Fig. 5, Fig. 6 show each of them successive 5-Gaussian peak, 6-Gaussian peak, 7-Gaussian peak, 8-Gaussian peak and 9-Gaussian peak fits of the 1PA spectrum of IR780 in the spectral range 18350–35980 cm^−1^, respectively. Fig. 7 shows the optimized structure of the molecule IR780 after applying three functionals.

Table 1, Table 2, Table 3, Table 4, Table 5 feature the results of the Gaussian-peak fitting procedures. Table 6 reports on the area under the curve (AUC) pertaining to the Gaussian fitting procedure. Table 7 shows the calculated oscillator strengths of the excited states S_n_ of IR780 in methanol. Table 8 contains results furnished from the data and the energy gap law formalism. Table 9, Table 10, Table 11 show the results of vertical transition calculations with different functionals and, finally, Table 12 reports the TPA cross-sections of IR780 estimated via quantum chemical calculations and three functionals.

**Table 1:** 5-Gaussian peak fit of the spectral bands of states S_n_ of IR780. $y_{0}$ is 95 ± 24 $M^{-1}{\text{cm}}^{-1}$. Reduced Chi-Sqr: 17627.6; R-Square: 0.9964; Adjusted R-Square: 0.9962.

Peak	$x_{c}\;\left(cm^{-1}\right)$	$w\;(cm^{-1})$	$A\;(M^{-1}cm^{-1})$
1	22500 ± 11	331 ± 15	1997 ± 79
2	23348 ± 32	1085 ± 22	3560 ± 58
3	26069 ± 10	374 ± 17	2675 ± 143
4	27173 ± 34	1150 ± 24	5109 ± 57
5	34615 ± 27	2580 ± 29	6928 ± 36

**Table 2:** 6-Gaussian peak fit of the spectral bands of states S_n_ of IR780. $y_{0}$ is 46 ± 23 $M^{-1}cm^{-1}$. Reduced Chi-Sqr: 10613.6; R-Square: 0.9979; Adjusted R-Square: 0.9977.

Peak	$x_{c}\;\left(cm^{-1}\right)$	$w\;(cm^{-1})$	$A\;(M^{-1}cm^{-1})$
1	21170 ± 67	461 ± 61	558 ± 40
2	22556 ± 19	483 ± 18	4325 ± 135
3	23821 ± 35	608 ± 36	3132 ± 80
4	26059 ± 10	343 ± 16	2159 ± 100
5	27035 ± 24	1269 ± 17	5269 ± 46
6	34596 ± 20	2549 ± 22	6996 ± 31

**Table 3:** 7-Gaussian peak fit of the spectral bands of states S_n_ of IR780. $y_{0}$ is 49 ± 18 $M^{-1}cm^{-1}$. Reduced Chi-Sqr: 8705.7; R-Square:0.9983; Adjusted R-square:0.9981.

Peak	$x_{c}\;\left(cm^{-1}\right)$	$w\;(cm^{-1})$	$A\;(M^{-1}cm^{-1})$
1	21155 ± 36	422 ± 0	567 ± 35
2	22545 ± 17	483 ± 12	4252 ± 129
3	23819 ± 29	633 ± 34	3166 ± 59
4	26048 ± 9	346 ± 15	2204 ± 103
5	27026 ± 24	1227 ± 19	5248 ± 45
6	34597 ± 106	2729 ± 80	6222 ± 34
7	34597 ± 260	1338 ± 98	850 ± 0

**Table 4:** 8-Gaussian peak fit of the spectral bands of states S_n_ of IR780. $y_{0}$ is 19 ± 14 $M^{-1}cm^{-1}$. Reduced Chi-Sqr: 3303.7; R-Square: 0.9994; Adjusted R-Square: 0.9993.

Peak	$x_{c}\;\left(cm^{-1}\right)$	$w\;(cm^{-1})$	$A\;(M^{-1}cm^{-1})$
1	21268 ± 48	564 ± 43	607 ± 22
2	22511 ± 8	437 ± 12	3572 ± 218
3	23732 ± 23	803 ± 56	3312 ± 82
4	26092 ± 9	452 ± 14	3960 ± 266
5	27309 ± 18	555 ± 25	2304 ± 194
6	27441 ± 126	1467 ± 82	2929 ± 186
7	34918 ± 44	27801 ± 74	6070 ± 35
8	33816 ± 76	1432 ± 86	1200 ± 0

**Table 5:** 9-Gaussian peak fit of the spectral bands of states S_n_ of IR780. $y_{0}$ is 19 ± 0 $M^{-1}cm^{-1}$. Reduced Chi-Sqr: 2690; R-Square: 0.9995; Adjusted R-Square: 0.9995.

Peak	$x_{c}\;\left(cm^{-1}\right)$	$w\;(cm^{-1})$	$A\;(M^{-1}cm^{-1})$
*1*	21282 ± 29	686 ± 0	609 ± 18
2	22544 ± 3	460 ± 3	3737 ± 34
3	23881 ± 0	816 ± 10	3390 ± 16
4	26091 ± 10	508 ± 7	5331 ± 100
5	27388 ± 19	655 ± 17	5071 ± 54
6	28765 ± 0	564 ± 44	1520 ± 121
7	29972 ± 111	686 ± 0	676 ± 65
8	32303 ± 0	285 ± 81	163 ± 36
9	34544 ± 10	2382 ± 18	7084 ± 13

**Table 6:** AUC from the fits of Section 2.2.

	AUC × 10^6^ (cm/mol)				
Excited State	5-peaks	6-peaks	7-peaks	8-peaks	9-peaks
S_2_	–	9.43	9.70	9.66	11.73
S_3_	2.44	55.99	55.08	45.36	45.49
S_4_	10.89	53.50	56.74	63.72	71.25
S_5_	3.28	24.54	25.63	74.36	74.36
S_6_	16.24	175.18	169.59	114.16	74.79

**Table 7:** Oscillator strengths of the upper excited states S_n_ of IR780 in methanol as a function of the number of Gaussian peaks used in the fitting.

	Experimental O.S.				
Excited state	5-peaks	6-peaks	7-peaks	8-peaks	9-peaks
S_2_	–	0.004	0.004	0.004	0.005
S_3_	0.011	0.024	0.024	0.020	0.020
S_4_	0.047	0.023	0.025	0.028	0.031
S_5_	0.014	0.011	0.011	0.032	0.032
S_6_	0.070	0.076	0.073	0.049	0.032

**Table 8:** γ, C and A calculated within the framework of the energy gap law. i.a.s., r.c.s. and c.o.b stand for indolyl-aromatic stretching, resonant-conjugated stretching and C-H aliphatic unsaturated trans out-of-plane bending, respectively.

Vibrational Modes		Energy gap law parameter		
Type	ℏω (cm^−1^)	γ	*C* (cm^−1^)	*A* × 10^12^ (s^−1^)
i.a.s.	1562	1.04	655	62.30
i.a.s.	1556	1.03	649	61.14
r.c.s.	1512	0.98	605	53.09
r.c.s.	1507	0.97	600	52.22
r.c.s.	1482	0.94	575	47.99
i.a.s.	1449	0.89	543	42.76
i.a.s.	1427	0.86	522	39.48
r.c.s.	1412	0.84	507	37.33
i.a.s.	1395	0.82	491	35.00
r.c.s.	1380	0.80	477	33.02
r.c.s.	1363	0.78	461	30.86
r.c.s.	1290	0.69	395	22.65
c.o.b	1255	0.64	365	19.29
c.o.b	1227	0.60	341	16.86
c.o.b	1168	0.53	293	12.44
c.o.b	1148	0.50	277	11.15
c.o.b	1118	0.46	254	9.39
c.o.b	1097	0.43	239	8.28
c.o.b	1079	0.41	226	7.41
c.o.b	1050	0.37	206	6.14
c.o.b	1031	0.35	193	5.40
c.o.b	992	0.30	168	4.09
c.o.b	976	0.27	158	3.63
c.o.b	893	0.17	112	1.82
c.o.b	788	0.03	65	0.62
c.o.b	741	-0.03	49	0.35
c.o.b	707	-0.08	39	0.22

**Table 9:** Results of the vertical transition calculation of the IR780 with functional PBE.

	Transition		
Excited state	Energy [eV]	Lambda [nm]	O.S.
	PBE-Gas-phase		
S_1_	2.083	595	2.026
S_2_	2.502	496	0.001
S_3_	2.823	439	0.021
S_4_	2.827	439	0.003
S_5_	3.007	412	0.096
S_6_	3.313	374	0.060

	PBE-Methanol		
S_1_	1.951	636	2.248
S_2_	2.541	488	0.001
S_3_	2.951	420	0.014
S_4_	2.962	419	0.008
S_5_	3.048	407	0.032
S_6_	3.305	375	0.096

**Table 10:** Results of the vertical transition calculation of the IR780 with functional PBE0.

	Transition		
Excited state	Energy [eV]	Lambda [nm]	O.S.
	PBE0- Gas-phase		
S_1_	2.214	560	2.248
S_2_	3.091	401	0.003
S_3_	3.595	345	0.035
S_4_	3.638	341	0.008
S_5_	3.680	337	0.001
S_6_	3.893	319	0.112

	PBE0-Methanol		
S_1_	2.058	603	2.411
S_2_	3.124	397	0.004
S_3_	3.685	337	0.009
S_4_	3.783	328	0.029
S_5_	3.803	326	0.010
S_6_	3.825	324	0.124

**Table 11:** Results of the vertical transition calculation of the IR780 with functional CAM-B3LYP.

	Transition		
Excited state	Energy [eV]	Lambda [nm]	O.S.
	CAM-B3LYP- Gas-phase		
S_1_	2.226	557	2.292
S_2_	3.614	343	0.014
S_3_	4.265	291	0.001
S_4_	4.316	287	0.116
S_5_	4.496	276	0.010
S_6_	4.522	274	0.011

	CAM-B3LYP-Methanol		
S_1_	2.051	605	2.422
S_2_	3.594	345	0.022
S_3_	4.248	292	0.143
S_4_	4.334	286	0.003
S_5_	4.628	268	0.021
S_6_	4.654	266	0.010

**Table 12:** Results of the TPA cross-sections of IR780 in the gas-phase and in methanol.

	TPA cross-sections (GM)					
	PBE0		PBE		CAM-B3LYP	
Excited State	Gas-phase	Methanol	Gas-phase	Methanol	Gas-phase	Methanol
S_1_	1	4	11	0.24	17	17
S_2_	2311	2267	4236	996	14654	7268
S_3_	754	1658	198	99	28131	25405182
S_4_	1406	25790	56	71	178559488	82380
S_5_	270	5648	1388	385	754507725	3167077
S_6_	490587	253451	195344	45648	6266	50341

Supporting files in [1] contain the raw data that substantiate Fig. 1, Fig. 2, Fig. 3, Fig. 4, Fig. 5, Fig. 6.

## 2 Experimental Design, Materials and Methods

**Scheme 1:**
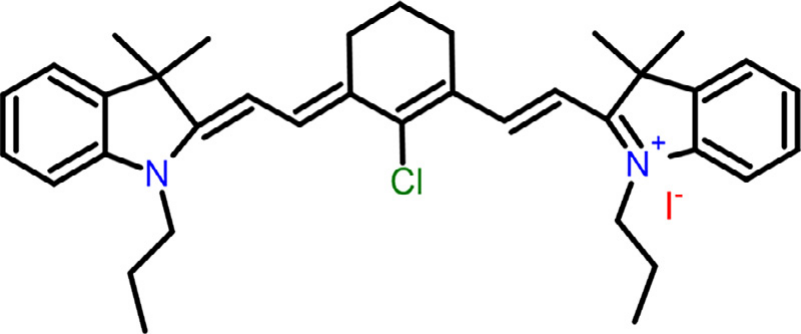
Molecular structure of the IR780.

In the experiment, dyes IR780, rhodamine B and rhodamine 6G were irradiated with a beam produced in an optical parametric amplifier (OPA) within a wavelength range of 850–1000 nm and the two-photon induced fluorescence was recorded to measure the two-photon absorption cross-section of IR780. The molecular structure of the cyanine IR780 is shown in Scheme 1.

The dyes and the methanol (HPLC-grade) used for the solutions employed in the measurements were purchased from Sigma-Aldrich. The concentrations utilized for the experiments were 2.25 × 10^−5^ M for IR780, 7.65 × 10^−6^ M for rhodamine B and 8.06 × 10^−5^ M for rhodamine 6G. The solutions were used immediately after preparation and, while the experiment was carried out, the temperature of the laboratory was kept at 20 ± 0.6 °C and the relative humidity at 45 ± 5%.

The laser system consists of a Ti:Sapphire mode-locked laser (Vitara-T, Coherent) seeding a regenerative amplifier (Legend-Elite DUO, Coherent) which, in turn, pumps an optical parametric amplifier (TOPAS OPA, Light Conversion). The output of the Legend (4.5 mJ per pulse, 1 kHz repetition rate, 80 fs, and pulse centered at 800 nm) is fed to the TOPAS where, in this case, radiation from 850 to 1000 nm is obtained. The power of the output beam from the OPA is controlled via OD filters and the polarization of the light at the sample is always vertical.

The excitation beam was expanded by a telescope and then steered to a microscope objective (Rossbach Kyowa) with NA = 0.1, 4x which focuses the light into the sample container, a sealed quartz cuvette with a 10 mm-optical path. The focal point was placed approximately 1 mm from the side wall of the cuvette. The pulse energy was varied between 0.002 and 0.35 μJ, producing peak intensities of ≈ 5.0 × 10^10^ to ≈ 7.9 × 10^12^ W cm^−2^ and controlled so the peak power never exceeded 8 × 10^12^ Wcm^−2^. The fluorescence from the dyes was collected at right angles with respect to the propagation vector of the excitation beam by an *f* = 50 mm, 1 in. diameter biconvex lens and focused to the entrance slit of an *f* = 300 mm Czerny-Turner monochromator coupled to a photomultiplier tube (PMT) (R12896, Hamamatsu Photonics). The output signal of the PMT was monitored with an oscilloscope (Tektronix 1102B-EDU). The peak power of the OPA was also independently monitored with a high-speed Si photodetector (DET110, 17.5 MHz bandwidth, 350–1100 nm, Thorlabs) also connected to the Tektronix oscilloscope and set to average over 64 samples.

Steady-state (linear) absorption and fluorescence spectra of the dyes were recorded for calibration purposes and were carried out in a Lambda-40 VU/vis and LS50B Luminescence spectrophotometers, both apparatus from Perkin-Elmer.

### 2.1 Quadratic dependence of the fluorescence for all the excitation wavelengths

In this section, a linear relationship between the Logarithm of the Integrated Fluorescence and the Logarithm of the peak intensity with slope 2 for each of the three compounds of interest (Rhodamine 6G, Rhodamine B and IR780) is shown. See Fig. 1. The raw data of the plots in Fig. 1 can be found in [1].

### 2.2 Deconvolution of the spectral bands of the states S_n_

The spectral bands were fitted using Gaussian functions described by the following equation

(1) $$y=y_{0}+A\exp\left[-0.5{\left(\frac{x-x_{c}}{w}\right)}^{2}\right],$$

where $y_{0}$ is the offset, $x_{c}$ is the centroid, w is the width and A is the amplitude of each peak. Table 1, Table 2, Table 3, Table 4, Table 5 and Fig. 2, Fig. 3, Fig. 4, Fig. 5, Fig. 6 show the parameters of the fit for the upper sates and the gaussian functions in the spectral range 18350 - 35980 cm^−1^, respectively.

A 10-peak fit of the spectral bands of the states S_n_ can be found in [2].

### 2.3 Experimental oscillator strength derived from the fitting of the upper excited states S_n_

The following equation was used to calculate the oscillator strength (O.S.) [3], [4]:

(2) $$F_{exp}=\frac{4ln10\varepsilon_{0}mc^{2}}{N_{a}e^{2}}\int \varepsilon \left(\bar{v}\right)d\bar{v},$$

where $\varepsilon_{0}$ is the vacuum permittivity (in C^2^/Nm^2^), *m* is the electron mass (in kg), *c* is the speed of light (in m/s), $N_{a}$ is Avogadro's number (6.02214076 × 10^23^ mol^−1^), *e* is the electron charge (in C) and the integral of $\varepsilon (\bar{v})$ is the area under the curve (AUC) of the peak-fitting by gaussian functions [5], [6]. The AUC is to be multiplied by a factor of 10 [3]. Tables 6 and 7 show the calculated AUC and O.S.’s of the excited states S_2_, S_3_, S_4_, S_5_ and S_6_ of IR780 in methanol, respectively.

### 2.4 Non-radiative decay properties of IR780 within the framework of the energy gap law

In this section, parameters γ (Potential Energy Surfaces shift), the matrix element of the vibrational coupling between electronic states (*C*) and the preexponential factor (*A*) from the energy gap law [2] are calculated (taking into account several vibrational modes (ℏω in Eq. (3)) of the cyanines).

From the slope (-6.0 × 10^−4^) and ΔE = 6786 cm^−1^ in [2], γ and *A* can be calculated via Eqs. (3) and (4), respectively:

(3) $$\gamma =-1-2.3\hbar \omega \left(slope+\frac{1}{4.6\Delta E}\right)$$

(4) $$A=\frac{\sqrt{2\pi }C^{2}}{\hbar \sqrt{\hbar \omega \Delta E}}$$

Table 8 shows these results for γ, *C* and *A*.

### 2.5 Computational results on the TPA cross-section of IR780

Quantum-mechanical calculations were carried out to gain further insight into the TPA properties of IR780. The molecular geometry of IR780 was optimized using Gaussian 09 [7]. The optimized structures (Fig. 7) were then used as input for TPA calculations (for six singlet excited states) carried out with GAMESS-US (The General Atomic and Molecular Electronic Structure System) [8]. TPA calculations were made in both the gas-phase and in a solvent (methanol) at the PBE0/6-31Gd, PBE/6-31Gd and CAM-B3LYP/6-31Gd levels of theory (CAM-B3LYP is the Coulomb Attenuating Method-Becke, 3-parameter, Lee-Yang-Parr; and PBE is the Perdew–Burke–Ernzerhof exchange-correlation functional).

The findings are summarized in Table 9, Table 10, Table 11. Two photon absorption cross-sections were calculated by using Eqs. (2) and (10) in [9].

## CRediT Author Statement

**Cesar A. Guarin:** Conceptualization, original draft preparation, methodology, investigation, software, data curation, validation, formal analysis; **Luis Guillermo Mendoza-Luna:** Original draft preparation, software, data curation, validation, methodology, investigation, formal analysis; **Emmanuel Haro-Poniatowski:** Supervision, funding acquisition, writing, investigation, writing - review and editing; **José Luis Hernández-Pozos:** Resources, funding acquisition, writing, investigation, writing - review and editing.

## Declaration of Competing Interest

The authors declare that they have no known competing financial interests or personal relationships which have, or could be perceived to have, influenced the work reported in this article.
